# Bridging language gaps: The role of NLP and speech recognition in oral english instruction

**DOI:** 10.1016/j.mex.2025.103359

**Published:** 2025-05-07

**Authors:** Parul Dubey, Pushkar Dubey, Rohit Raja, Sapna Singh Kshatri

**Affiliations:** a Symbiosis Institute of Technology, Nagpur Campus, Symbiosis International (Deemed University), Pune, India; b Department of Management, Pandit Sundarlal Sharma (Open) University Chhattisgarh, India; c Department of Information Technology, School of Studies (Engineering and Technology), Guru Ghasidas Vishwavidyalaya (A Central University), India; d Department of Artificial Intelligence, Shri Shankaracharya Institute of Professional Management and Technology, Raipur, India

**Keywords:** Face multimodal natural language processing (NLP), Speech recognition, Hidden markov models (HMMs), Oral english instruction, Deep learning, Multimodal NLP and Speech Recognition

## Abstract

The Natural Language Processing (NLP) and speech recognition have transformed language learning by providing interactive and real-time feedback, enhancing oral English proficiency. These technologies facilitate personalized and adaptive learning, making pronunciation and fluency improvement more efficient. Traditional methods lack real-time speech assessment and individualized feedback, limiting learners' progress. Existing speech recognition models struggle with diverse accents, variations in speaking styles, and computational efficiency, reducing their effectiveness in real-world applications. This study utilizes three datasets—including a custom dataset of 882 English teachers, the CMU ARCTIC corpus, and LibriSpeech Clean—to ensure generalizability and robustness. The methodology integrates Hidden Markov Models for speech recognition, NLP-based text analysis, and computer vision-based lip movement detection to create an adaptive multimodal learning system. The novelty of this study lies in its real-time Bayesian feedback mechanism and multimodal integration of audio, visual, and textual data, enabling dynamic and personalized oral instruction. Performance is evaluated using recognition accuracy, processing speed, and statistical significance testing. The continuous HMM model achieves up to 97.5 % accuracy and significantly outperforms existing models such as MLP-LSTM and GPT-3.5-turbo (*p* < 0.05) across all datasets. Developed a multimodal system that combines speech, text, and visual data to enhance real-time oral English learning.

• Collected and annotated a diverse dataset of English speech recordings from teachers across various accents and speaking styles.
• Designed an adaptive feedback framework to provide learners with immediate, personalized insights into their pronunciation and fluency.

## Graphical abstract

**Figure:**
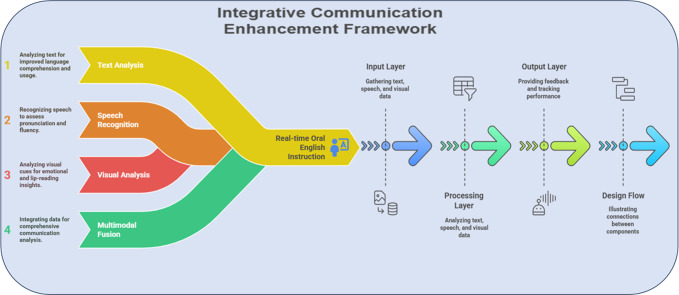

Specifications table

**Table:** 

Subject area:	Computer Science
More specific subject area:	Multimodal NLP and Speech-Based Learning
Name of your method:	Multimodal NLP and Speech Recognition
Name and reference of original method:	[1] K. Kandji, C. Ba, and S. Ndiaye, State-of-the-Art Review on Recent Trends in Automatic Speech Recognition. 2024, pp. 185–203. 10.1007/978–3–031–63,999–9_11. [2] G. Antoniadis, S. Granger, O. Kraif, C. Ponton, J. Medori, and V. Zampa, “Integrated Digital Language Learning,” in Springer eBooks, 2009, pp. 89–103. 10.1007/978–1–4020–9827–7_6.
Resource availability:	Datasets: Custom multimodal dataset of 882 annotated speech recordings- 10.6084/m9.figshare.28703093 “Speaker Recognition - CMU ARCTIC,” Kaggle, Nov. 21, 2022. https://www.kaggle.com/datasets/mrgabrielblins/speaker-recognition-cmu-arctic “LibriSpeech ASR corpus,” Kaggle, Oct. 16, 2023. https://www.kaggle.com/datasets/pypiahmad/librispeech-asr-corpus

## Background

The Technology has been transforming the world, impacting nearly every aspect of life. When we turn toward education, this technological advancement becomes more than remarkable. In effect, this has produced new ways of learning—language learning, most notably. The integration of Natural Language Processing (NLP) and speech recognition technologies has become one of the most perceptively effective means to assist language learning, especially in oral English practices [1].

Traditional teaching techniques of oral English are frequently based around repetitive drills, and instructor interactions and regulation form an issue, particularly where these require labor-intensive interaction with instructors [2]. Digital language learning tools have arguably done more to enable personalized and flexible learning experiences [3]. But despite the strides made, sophisticated, higher-touch methods are still required for a more in-depth learning experience that meets learners where they need to be met. This is where multimodal NLP, which involves text and speech in equal measure along with other more complete forms of input, is a solution to help focus requirements for language learning [[4], [22], [23]]. When paired with top-notch speech recognition technology, they can provide dynamic and interactive learning platforms that mirror real-world communication interactions. By combining the two uses, not only is language instruction more accurate and faster, but learner engagement and motivation are also significantly increased [5,6].

**Fig. 1:**
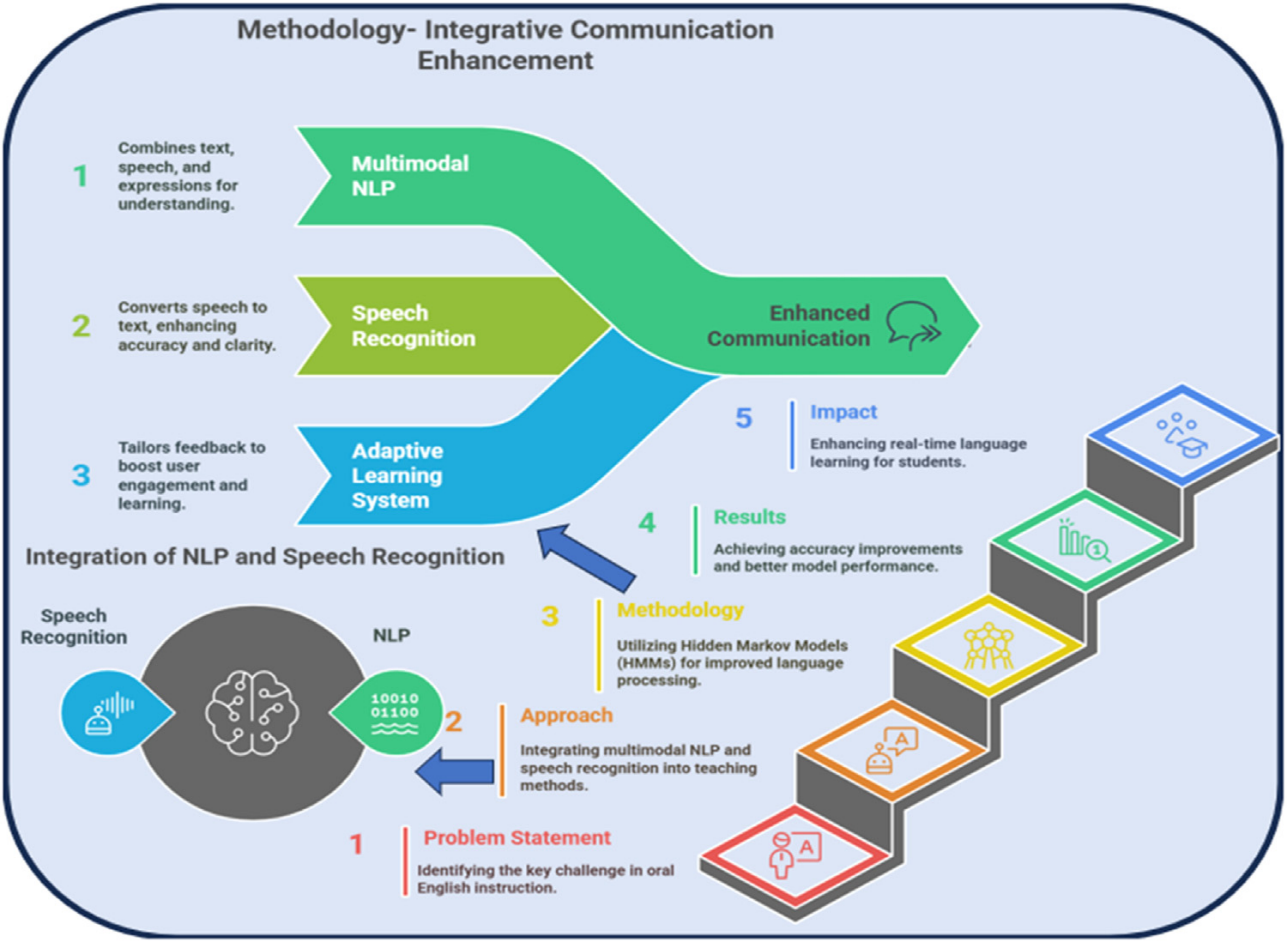
Conceptual framework of integrative communication enhancement using multimodal NLP and speech recognition for real-time language learning.

This paper explores multimodal NLP and speech recognition technologies for innovative oral English instruction. This will be an overview of those techniques, their current state, and the types of challenges they are ill-suited to address when applied to language learning and more advanced deep learning methods that can solve these issues. Thus, we hope to improve the efficiency of oral English practice and establish a foundation for further studies on this topic. In the next sections, we will briefly describe the theoretical background of multimodal NLP as well as speech recognition and explain how these features can be integrated into language learning systems from the more important evidence stemming from recent studies. The figure of the abstract can be seen in Fig. 1.

The novelty of the current research is that it combines speech, text, and visual lip movement for real-time provision of multimodal feedback for oral English instruction to improve the quality of feedback—an area that has rarely been explored in existing educational speech systems. Our approach departs from conventional unimodal models to integrate speech recognition via audio with visual features and context-aware NLP to improve both recognition performance and learner engagement. Furthermore, continuous and semicontinuous Hidden Markov Models (HMMs) are employed, providing a flexible trade-off between precision and speed, which is necessary for a real-time system. Moreover, the incorporation of a Bayesian adaptive learning mechanism, based on user-specific feedback data, provides cases of dynamic-adjustable paths for the AOT learning process, moving more ahead than existing automated approaches for oral language instruction. Validated across three datasets with statistical significance, these innovations are marked as a significant move in the space of AI language learning technologies.

Here are key technical contributions of this paper:

1. Multimodal Integration for Adaptive Feedback: Originally designed to combine speech, text, and visual lip movement data, the system delivers real-time, personalized feedback to enhance oral English instruction. This core contribution remains central to the model's learner-centric approach.
2. High-Accuracy Speech Recognition with Optimized HMMs: The use of both continuous and semicontinuous Hidden Markov Models (HMMs) enables a balance between recognition accuracy and processing speed, achieving up to 97.5 % accuracy in close-testing scenarios.
3. Optimized Real-Time Processing for Language Learning: The implementation of semicontinuous HMMs ensures faster feedback delivery, making the system practical for live classroom and mobile applications.
4. Bayesian Adaptive Feedback Mechanism (New):A novel Bayesian updating framework has been incorporated to personalize feedback dynamically, using probabilistic reasoning based on learner-specific pronunciation and fluency data.
5. Cross-Dataset Benchmarking with Statistical Validation (New): The system has been validated across three datasets—our custom teacher corpus, CMU ARCTIC, and LibriSpeech Clean—and significantly outperforms baseline models like MLP-LSTM and GPT-3.5-turbo with p-values < 0.05, confirming its robustness and generalizability.

The NLP and speech recognition-related technologies have recently been copiously embedded in the domain of language education, including oral English teaching. Studies have shown the pros and cons of these technologies being used in educational contexts. The following literature review focuses on challenges, applications, and directions of NLP and speech recognition technologies concerning language learning.

Drawing on four learning theories (knowledge construction, inquiry-based learning, dialogic teaching, and the zone of proximal development), researchers propose several scaffolding tutoring solutions. They created and iteratively improved upon a seven-dimensional rubric that could guide the assessment of the scaffolding process for both quantitative and qualitative evaluations. They discovered a lot of potential in GPT-4 experiments with LLMs to execute instruction directions and achieve self-paced, diverse, and disparate learning in various populations of students. The best accuracy was 80.5 percent on GPT-3 with 3-shot inference. 5-turbo-1106 model [7]. With the same model, the F1 score is 79.5. We will compare our model with these research results.

Several other research studies have emerged, focusing on key challenges to integrating NLP and speech recognition technologies into language education [8]. A significant challenge is the extraction and segmentation of features from multimodal data, which is essential for enhancing speech recognition accuracy and delivering valuable feedback to learners [[26], [27]]. According to studies, whilst feature extraction errors have a high impact on model performance, this reduces NLP-driven learning platforms efficiency [28]. Moreover, researchers have identified policy constraints, technological limitations, and educator resistance as obstacles to the widespread adoption of NLP in university teaching. Institutional policy frequently trails technology: the implementation of sophisticated learning instruments can be thwarted by policy [9]. Additionally, technological limitations such as computational constraints and the absence of robust infrastructure also hinder the smooth integration of NLP-based applications. Moreover, opposition from teachers, mostly because of unaccustomedness or doubt about the efficiency of such technologies in comparison to conventional teaching styles, posed a challenge.

However, various challenges exist, but NLP and speech recognition technologies are showing great promise in enhancing oral English teaching. Other studies have investigated the applications of neural network technologies, including Speech ace and Rosetta Stone, both of which have demonstrated their efficiency in developing auditory and speaking abilities [10]. These services use NLP to break down the spoken words and provide feedback so the learners can develop their pronunciation, fluency, and comprehension.

In recent years, using machine learning techniques like MLP-LSTM (Multilayer Perceptron-Long Short-Term Memory) networks to extract features or transfer sequences has made spoken language transformation more accurate [8]. At the same time, Word Error Rate (WER) has become a way to measure success. (These improvements can be extremely useful in, e.g., self-paced learning environments, where learners may prefer automated, immediate feedback without an instructor always being present.) Moreover, speech recognition technology has been used to improve English proficiency and communication skills, which provide a more interactive learning experience [11].

Educational institutions have already started using speech recognition and NLP technologies in several different settings. NLP technology is increasingly accepted in academic institutions, as interviews with university teachers show; according to empirical research, 80 % of the interviewed teachers have already used NLP technology in the classroom [9]. These technologies have also been usefully embedded into computer-assisted language learning (CALL) systems, which allow learners to analyze their pronunciation, fluency, or practice conversations with a virtual agent [12]. This uses NLP to analyze speech patterns and understand comprehension levels, providing users with tailored feedback. These innovations demonstrate that this potential is not limited to traditional language instruction but also caters to domain-oriented learning applications in addition to language-specific subject knowledge.

Research shows that voice recognition technology will get better with the help of more advanced deep learning methods, new algorithmic architectures, and better ways to process data from multiple sources [13]. With this increase in accuracy, speech recognition can better adapt to different languages, dialects, and accents by using more and more advanced deep learning architectures, like those based on transformers for NLP tasks.

It is also expected that adding ideas from corpus linguistics to NLP-based language learning tools will make them more useful for education [14]. By leveraging extensive linguistic databases, future NLP systems can offer context-aware, personalized learning experiences that align with learners' unique needs. These improvements will likely result in more powerful and effective tools for technology-enhanced language learning, ultimately bridging the gap between artificial intelligence and human-like language instruction. Research gaps are listed as follows:

• Existing speech recognition models struggle with accent variations and speaking speeds, while balancing accuracy and real-time processing remains a challenge.
• Current systems primarily focus on text and speech, neglecting visual cues like lip movements and facial expressions that enhance learning.
• Many platforms fail to support diverse linguistic populations, and educator hesitation due to training gaps and reliability concerns slows adoption.

Traditional oral English instruction often lacks personalized, real-time feedback, limiting learner engagement and overall effectiveness. Conventional teaching methods, including repetitive drills and instructor-led sessions, fail to address individual learner needs effectively. Furthermore, language learning platforms often struggle with diverse accents, speech patterns, and pronunciation variations, making them less adaptive to real-world linguistic diversity. The integration of Natural Language Processing (NLP) and speech recognition technologies has the potential to bridge this gap by offering automated, adaptive, and interactive language learning solutions. However, the implementation of these technologies faces challenges related to feature extraction, accuracy, processing efficiency, and educator adoption. This research aims to explore how multimodal NLP and speech recognition models can improve oral English instruction, particularly by enhancing speech recognition accuracy and real-time feedback mechanisms. This gap is effectively addressed through a multimodal approach, which enriches feedback and enhances recognition accuracy by incorporating speech, text, and visual signals.

## Method details

A complete method is needed to improve the use of multimodal NLP instruction along with speech recognition for speaking English practice. This method includes data collection, system architecture, model training, and evaluation processes. The data collection phase begins with the compilation of multimodal data, which includes gathering diverse text data from sources such as textbooks, online articles, conversation transcripts, and educational content. In addition, a considerable number of recordings are compiled for both native and nonnative English speakers to provide an abundant variety in accents, dialects, and speaking styles so as to attain a well-balanced data pool. Visual data is captured, and appropriate video recordings are then stored, e.g., lip movements. This data is then manually annotated with linguistic and phonetic features: part-of-speech tags, semantic roles and a broad transcription of the text. It also labels textual emotion tags, fluency scores, and additionally semantic content to better allow the model to give more nuanced feedback.

We chose a multimodal approach (speech, text, and visual features predicting lip movement) since unimodal systems were insufficient, especially within the domain of oral English instruction [[32], [35], [36]]. Speech recognition systems trained on audio alone are known to perform poorly when dealing with accents, pronunciation ambiguities, and background noise during education sessions. This is further complemented by visual cues such as lip movement and facial expressions that can significantly reduce confusion in speech disambiguation, particularly when dealing with phonetically similar words. In addition, text-based NLP analysis allows for greater context, which can provide more intelligent feedback. Combining these three modalities together leads to better recognition accuracy, noise insensitivity, and richer domain-adaptation feedback that are well-suited for personalized language learning environments.

The system architecture proposed is designed to efficiently process multimodal input, i.e., the current implementation starts with a text processing module that uses Natural Language Processing (NLP) techniques—tokenization, part of speech tagging, Named Entity Recognition (NER), syntactic parsing, etc.—for semantic comprehension of textual content. Using a good speech recognition module, spoken language is transcribed to text through various methods such as noise removal and speaker normalization (to increase transcription accuracy). Just like other computer vision technologies—for visual processing, we employ computer vision utilizations to understand the inputs through visuals, i.e., images and videos that incorporate lip movement or facial expression in case of highly efficient speech recognition combined with emotion detection. This information then flows through to a (modality-wise) fusion layer that fuses the multimodal features and uses attention gates on each modality in context. The data is used to train deep learning models, and the most commonly used are Convolutional Neural Networks (CNNs) for visual data or Recurrent Neural Networks (RNNs) and transformers for text and speech; these include GPT-2, and BERT itself can be tuned accordingly. The algorithms used in this process are explained in upcoming sections. The HMM-based models used five hidden states per phoneme, optimized through grid search during training.

The model training part would also include preprocessing and normalization of the input text, audio, and visual data, such as lowercase, stop word removal, and stemming functions for the input text, and noise reduction, segmentation, and feature extraction (e.g., MFCCs) from audio. We perform data augmentation to expand the size and diversity of images in the dataset, thereby enhancing the generalization properties of the model. It has a variety of ways to be trained with labelled audio data using supervised learning, and it can use Connectionist Temporal Classification (CTC) [18] for variable-length sequences. We train our multimodal NLP model with a mix of supervised and unsupervised learning on task-specific datasets (e.g., conversation transcripts or language learning exercises) to avoid overfitting using cross-validation.

**Fig. 2:**
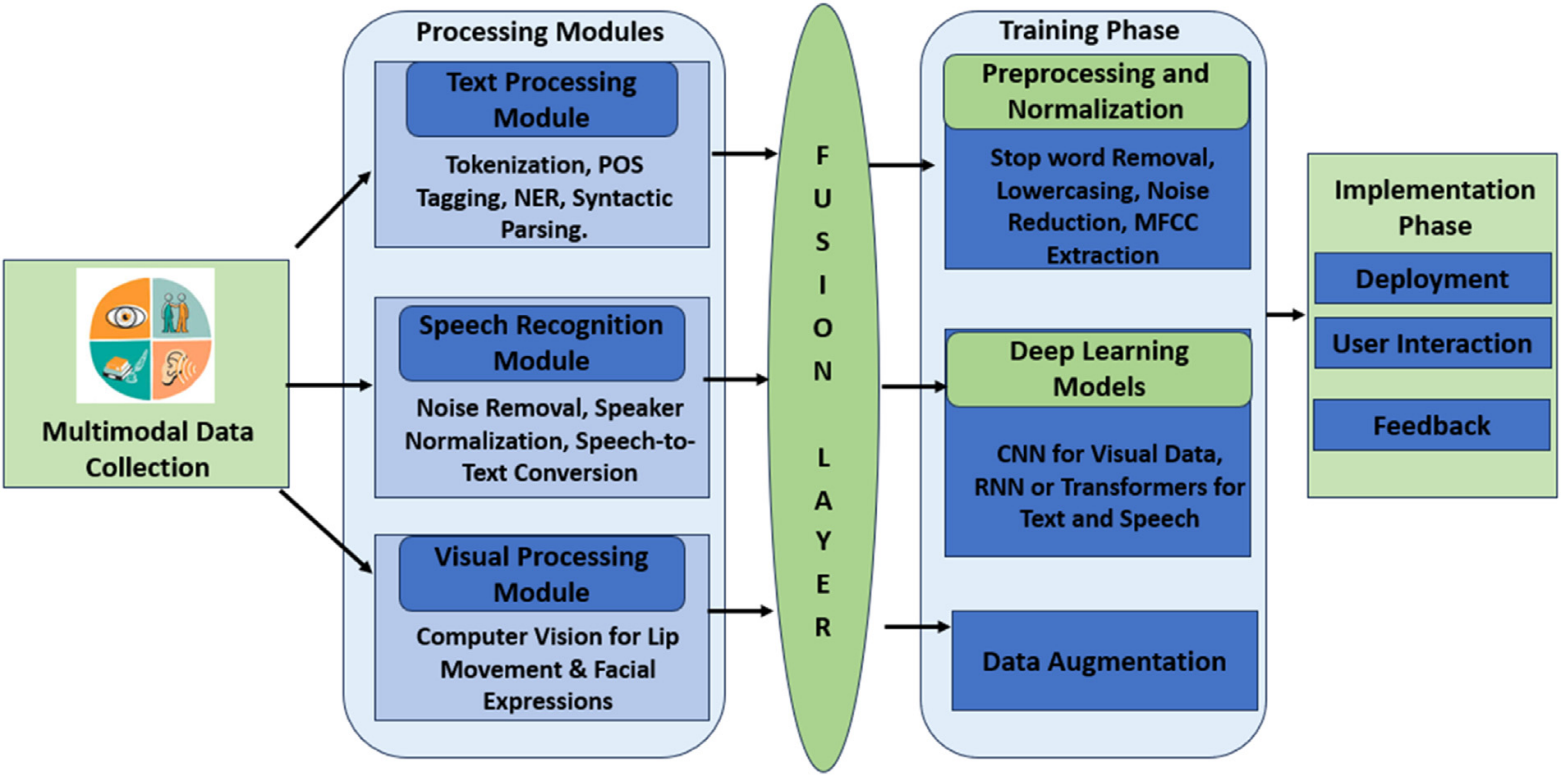
Architecture of the proposed model.

Lastly, in phase 4, which is the implementation stage, we deploy our project, making it accessible to a larger audience. The tool has been built with an easier learning curve and live feedback for users to understand, and each unit is followed by quick quizzes and practice sessions, tracking the progress. Through user feedback and app performance data, the system will be iteratively improved upon using a continuous improvement approach that responds to learner needs with more updates and new features in line with advances in technology. In this work, we use a new method that is able to improve the effect of oral English teaching greatly by borrowing multimodal NLP and speech recognition techniques. Advanced deep learning techniques are integrated so they are robust and accurate in providing personalized feedback, which results in improved learner outcomes. The flow chart for the methodology applied can be seen in Fig. 2.

### Dataset

This collection was developed especially to support multimodal research on NLP and speech recognition for the practice of oral English. This dataset spans over 882 different participants, increasing the scope and richness of data available for researchers or developers.

The dataset contains audio recordings of 882 professional oral English teachers, among whom male and female participants are involved. This dataset contains utterances spoken by participants with a wide variety of speech patterns and accents, which adds to the diversity in these types of voice datasets. This enables each anonymous participant to engage in a 71.5 min recording session, resulting in a substantial number of records per person. This larger recording time window allows for a deeper analysis and training of speech recognition models. Participants were in contact with 1755 spoken English words spread over a set of 3,410 sentences. This contains more sentences and vocabulary diversity and would further enrich the spoken English in this dataset, as a matter of well-trained large-scale language models pretraining from better resources. The stereo recordings are made using a MAYA MKH 8020 microphone at a sampling rate of 16 kHz with a single track and a quantification length of 16 bits at a bit rate of 256 kbps. It records the talks in making use of Pulse Code Modulation (PCM) record file format, which means that this is without a doubt so obvious and high-quality was understood.

Each sentence has detailed word-level annotations that offer exact information, both linguistically and phonetically. Since the dataset is meticulously annotated, it can cater to different tasks in NLP ranging from phonetic recognition to syntactic parsing [7]. For applicable instances, emotion tags have been used as well to help in the future with creating tools that can understand human emotions from their voice.

We perform various preprocessing operations on the data before using it in speech recognition and NLP models. We extract features like pitch trajectories and Mel-frequency cepstral coefficients (MFCCs) from these audio recordings. These kinds of factors are important because they allow for a correct and realistic mapping of phonetic aspects that contrast tone in language [15,16]. The dataset also accommodates various learning strategies, such as choosing course hours at will, a 30-day structured learning plan, and intensive review sessions with fill-in-the-blank multiple-choice questions. The main purpose of including them is to improve language learning and memorization [17].

We expect it to be useful in developing and evaluating multimodal NLP and speech recognition systems. This can range from research activities such as training deep learning models to experimental NLP tasks [18,19]. Our extensive dataset can be used to further state-of-the-art language technology, ranging from the richness and size of its participants to detailed annotations with a large variety. Table 1 illustrates the transformations applied to both textual and audio data, improving consistency, quality, and usability for the NLP and speech recognition models.

**Table 1:** Data before and after preprocessing.

Data Attribute	Before Preprocessing	After Preprocessing
Total Sentences	3410	3410 (filtered for duplicates and noise)
Total Words	1755	1655 (lowercased, stop words removed, duplicates reduced)
Word Annotations	Raw, untagged	Tagged with parts of speech, fluency scores, and semantic roles
Audio Noise Level	High variability, includes background noise	Reduced noise, filtered using noise-reduction techniques
Accent and Dialect Variability	Wide range, unbalanced	Balanced through normalization and accent filtering
Sampling Rate	16 kHz	16 kHz (unchanged)
Pitch and Frequency Variability	Unprocessed	Smoothed and normalized, including Mel-Frequency Cepstral Coefficients (MFCCs)
Emotion Tags	None	Added manually or via automated tagging
Format	Pulse Code Modulation (PCM)	Converted to standardized format for consistency in analysis
Data Augmentation	Not applied	Augmented with pitch shift, speed variation, and background noise addition

To assess the generalizability and robustness of the proposed multimodal speech recognition model, two publicly available benchmark datasets were added to our custom dataset (which contains 882 annotated speech recordings) for use in this work. The first is the CMU ARCTIC Speech Database [42], a widely used database for speaker recognition and speech synthesis research, which consists of a phonetically balanced corpus. It consists of recordings from several male and female native English speakers with an American accent. The second is the LibriSpeech ASR Corpus (Clean Subset) [43] that features excellent quality read English speech extracted from audiobooks, creating an actual standard benchmark for automatic speech recognition systems in clean acoustic conditions. The availability of these datasets supports stringent cross-dataset comparisons and also allows for a statistical characterization of how well a given model performs under diverse linguistic and acoustic conditions.A detailed summary of the datasets used for training, validation, and benchmarking purposes—including speaker demographics, speech types, and intended roles in the study—is provided in Table 2.

**Table 2:** Description of datasets used for model evaluation.

Dataset	Description	Source	No. of Speakers	Speech Type	Purpose in Study
Custom English Teachers Dataset	Annotated recordings of professional English teachers from diverse linguistic backgrounds	Our Dataset (882 samples)	882	Read and spontaneous	Model training, fine-tuning, baseline evaluation
CMU ARCTIC Speech Database	Phonetically balanced speech for speaker recognition and synthesis	[42]	7 (US English)	Scripted (phonetically rich)	Cross-accent validation, ASR benchmarking
LibriSpeech ASR (Clean)	Read English speech from audiobooks with clean acoustic conditions	[43]	40+	Read (audiobook-derived)	Robustness and generalization testing

### Multimodal NLP and speech recognition

The multimodal NLP and speech recognition system relies on certain essential mathematical computations [[13], [14], [15]]. The computations encompass several components of the system, such as feature extraction, voice recognition, emotion detection, and adaptive learning. The following are the essential mathematical elements:

### Feature extraction

Mel-Frequency Cepstral Coefficients (MFCCs) are a popular feature used in speech recognition that represents the short-term power spectrum of a sound [24,25]. The calculation involves several steps:

Pre-Emphasis Filter can be represented by Eq. (1)

(1) y[n]=x[n]−α.x[n−1]

where x[n] is the input signal, y[n] is the output signal, and α is the pre-emphasis coefficient, typically around 0.95 [19].

Framing and Windowing: The signal is divided into overlapping frames. Each frame is multiplied by a Hamming window, which is shown in Eq. (2):

(2) $$\left(n\right)=0.54-.46\;\cos\left(\frac{2\pi n}{N-1}\right)$$

where N is the frame length.

Fast Fourier Transform (FFT) is applied to each frame to obtain the frequency spectrum [20], this is shown in Eq. (3):

(3) $$X\left[k\right]=\sum_{n=0}^{N-1}x\left[n\right].e^{-j.2\pi kn/N}$$

where X[k] is the FFT result.

Mel Filter Bank,the power spectrum is passed through a Mel filter bank as represented by Eq. (4):

(4) $$S_{m}=\sum_{k-f_{min}}^{f_{max}}{\left|X\left[k\right]\right|}^{2}.H_{m}\left[k\right]$$

where $H_{m}\left[k\right]$ represents the m-th triangular filter in the Mel filter bank.

Logarithm of Filter Bank Energies is shown in Eq. (5).

(5) $$\log S_{m}=\log\left(S_{M}\right)$$

Discrete Cosine Transform (DCT), the final MFCCs are obtained by applying the DCT to the log filter bank energies, as given by Eq. (6).

(6) $$MFCC_{n}=\;\sum_{m=1}^{M}\log S_{m}\cos\left(\frac{\pi n\left(2m+1\right)}{2M}\right)$$

where M is the number of filters in the Mel filter bank.

Pitch estimation can be performed using the autocorrelation method.Autocorrelation Function is represented by Eq. (7).

(7) $$R\left(\tau \right)=\sum_{n=0}^{N-1}x\left[n\right].x\left[n-\tau \right]$$

where R(τ) is the autocorrelation function and τ is the lag.

The pitch period $T_{0}$ is found by identifying the peak in the autocorrelation function corresponding to the pitch frequency, as shown in Eq. (8).

(8) $$f_{0}=\frac{1}{T_{0}}$$

### Speech recognition

Hidden Markov Models (HMMs) are commonly used in speech recognition. For the proposed model, the number of hidden states was set to 5 HMM per phoneme, a standard in phoneme-level modeling in speech recognition. Specifically, the HMM includes five hidden states to represent the evolution of speech signals across time for each unique phoneme unit, in the form of onset, steady-state, and offset properties. A grid search was performed using the number of states per phoneme ranging from 3 to 7 through cross-validation to empirically determine the choice. The five states produced the best trade-off between recognition accuracy and computational cost. This configuration was uniform in continuous and semicontinuous HMM implementation in the study. They involve the following calculations:

Forward Algorithm: Given an HMM with states S1, S2,…,SN and observations O1,O2,…,OT, represented by Eq. (9):

(9) $$\alpha_{t}\left(i\right)=\left\lceil \sum_{j=1}^{N}\alpha_{t-1}\left(j\right).a_{ji}\right\rceil .b_{i}\left(O_{t}\right)$$

where αt(i) is the probability of being in state Si​ at time t and observing O1, O2, …, Ot, aji is the state transition probability, and bi(Ot) is the observation likelihood.

The Viterbi algorithm is used to find the most probable state sequence by formula (10).

(10) $$\delta_{t}\left(i\right)=max_{1\leq j\leq N}\left[\delta_{t-1}\left(j\right).a_{ji}\right].b_{i}\left(O_{t}\right)$$

where $\delta_{t}\left(i\right)$ represents the maximum probability of any state sequence ending in state $S_{i}\;$at time t

### Emotion recognition

Gaussian Mixture Models (GMMs) are used to model the distribution of speech features for different emotions [21]. GMM Probability Density Function is denoted by Eq. (11):

(11) $$p\left(x|\gamma \right)=\sum_{k=1}^{K}w_{k}.N\left(x;\mu_{k},\sum k\right)$$

where x is the feature vector, K is the number of Gaussian components, $w_{k}$ ​ are the mixture weights, µk ​ are the mean vectors, and Σk​ are the covariance matrices [29,30].

Expectation-Maximization (EM) Algorithm: Used to estimate the parameters of the GMM denoted by Eq. (12)-(14).

(12) $$w_{k}^{new}=\frac{1}{N}\sum_{I=1}^{N}\gamma_{K}\left(i\right)$$

(13) $$\mu_{k}^{new}=\frac{\sum_{i=1}^{N}\gamma_{K}\left(i\right).x_{i}}{\sum_{i=1}^{N}\gamma_{k}\left(i\right)}$$

(14) $$\sum_{k}^{new}=\frac{\sum_{i=1}^{N}\gamma_{K}\left(i\right).\left(x_{i}-\mu_{k}^{new}\right){\left(x_{i}-\mu_{k}^{new}\right)}^{T}}{\sum_{i=1}^{N}\gamma_{k}\left(i\right)}$$

where γk(i) is the posterior probability that x_i_ ​ was generated by the k-th Gaussian component [31].

### Adaptive learning and feedback mechanisms

**Fig. 3:**
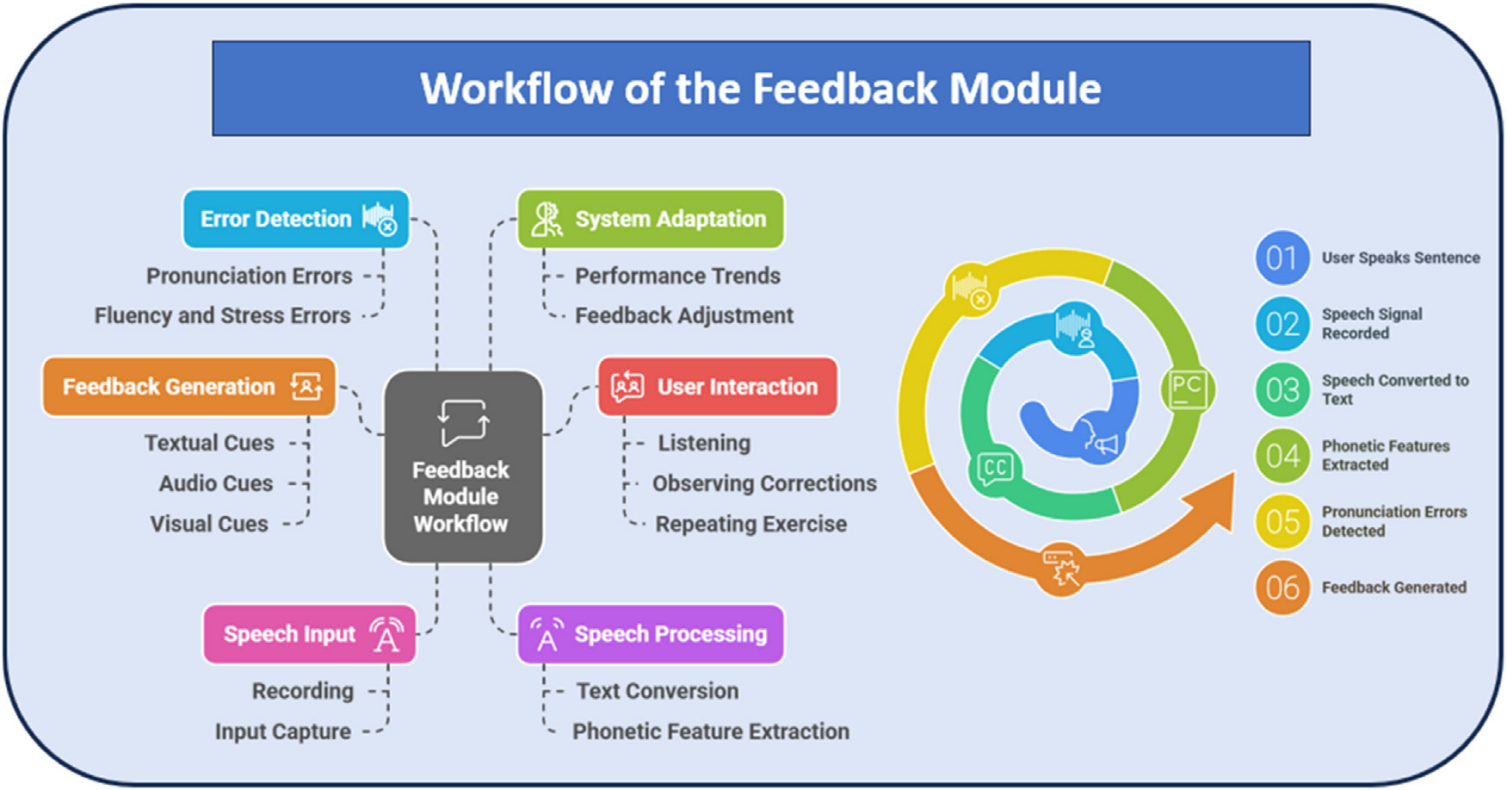
Workflow of the feedback module for speech recognition-based language learning.

The adaptive feedback mechanism updates a learner's pronunciation knowledge using Bayesian inference. Let θ represent the learner’s latent pronunciation skill level (e.g., correct production of /θ/ sound), and D be the observed data (e.g., pronunciation score and fluency score from the system). For example, if a learner consistently mispronounces “the” as “de” (as shown in Table 8), the observed data D would reflect low phoneme match scores. Fig. 3 shows the workflow of the feedback module.

The posterior knowledge estimate is computed using Bayes’ theorem,as given by Eq. (15):

(15) $$P\left(\theta |D\right)=\frac{P\left(D|\theta \right).P\left(\theta \right)}{P\left(D\right)}$$

P(θ) is the prior probability based on pre-assessment or initial model scores.

P(D | θ) is the likelihood of observing the learner’s mispronunciation given their knowledge level.

P(θ | D) is the updated estimate of the learner’s knowledge state.

For instance, in our dataset, initial pronunciation success rate (prior) for a phoneme like /ð/ was 0.6. After 5 trials with observed success in 4 cases (fluency and pronunciation score > 85 %), the posterior becomes equation(16):

(16) $$P\left(\theta |D\right)\propto \theta^{4}\cdot {\left(1-\theta \right)}^{1}$$

which is a beta distribution Beta(5, 2) after conjugate prior update. This updated belief helps the system decide whether the learner has mastered the phoneme or needs targeted feedback again. Such Bayesian tracking enables individualized difficulty adjustment for repeated incorrect segments, making the learning process more personalized and efficient.

To provide clarity on how the learner’s knowledge state is updated in our adaptive system, we present the complete mathematical derivation of the Bayesian update rule as applied to pronunciation performance data.Let θ denote the learner’s *true but unknown* pronunciation ability (e.g., probability of correctly pronouncing /ð/).

We assume:

• Each learner attempt is an independent Bernoulli trial (correct or incorrect pronunciation).
• The prior distribution of θ\theta is modeled as a Beta distribution is shown in Eq. (17):
  (17) $$P\left(\theta \right)=Beta\left(\alpha ,\beta \right)=\frac{\theta^{\alpha -1}{\left(1-\theta \right)}^{\beta -1}}{\beta \left(\alpha ,\beta \right)}$$

where Beta(α,β) is the Beta function.

After observing data $D=\{x_{1},x_{2},\ldots .,x_{n}\}$ where each $x_{i}\in \left\{0,1\right\}$ is a learner’s pronunciation result:

• Let s= $\sum x_{i}$: number of correct pronunciations,
• Let f=n - s: number of incorrect pronunciations.

The likelihood of the observed data given θ is shown is Eq. (18):

(18) $$PP\left(D|\theta \right)=\theta^{s}{\left(1-\theta \right)}^{f}$$

Applying Bayes’ theorem we obtain following Eqs. (19) and (20):

(19) $$P\left(\theta |D\right)=\frac{P\left(D|\theta \right)\cdot P\left(\theta \right)}{P\left(D\right)}\propto \theta^{s}{\left(1-\theta \right)}^{f}\cdot \theta^{\alpha -1}{\left(1-\theta \right)}^{\beta -1}$$

(20) $$P\left(\theta |D\right)\propto \theta^{s+\alpha -1}{\left(1-\theta \right)}^{f+\beta -1}$$

Thus, the posterior distribution is also a Beta distribution can be then represented by Eq. (21):

(21) P(θ|D)=Beta(α+s,β+f)

Example from Our Data: Suppose the prior is Beta(2,2) (i.e., we assume moderate prior uncertainty). If the learner makes 5 pronunciation attempts and succeeds in 4, then we obtain following Eqs. (22) and (23) for applying these values:

(22) s=4,f=1⇒P(θ|D)=Beta(2+4,2+1)=Beta(6,3)

The posterior mean estimate of θ is:

(23) $$E\left[\theta |D\right]=\frac{6}{6+3}=0.667$$

This updated belief indicates that the learner now has an estimated 66.7 % chance of correctly pronouncing this phoneme and should receive targeted practice until crossing a threshold (e.g., >85 %).

Adaptive Learning Rate:

To adjust the learning rate in response to learner performance Eq. (24) was used.

(24) $$\varphi_{t+1}=\varphi_{t}.\left(1-\frac{\Delta L_{t}}{L_{t}}\right)$$

where $\varphi_{t}$​ is the learning rate at time t, Lt is the loss function, and ΔLt is the change in loss [33].

## Method validation

System performance varies by test configuration. The following section discusses them.

Closed Testing: Data entered by the participants, used for training as well as testing. It's essentially the same as above, but it tests if a system knows who users are and how they talk.

Open Testing: This method utilizes participant data not present in the test's training set. This measures the solution's generality to distinguish between a random new voice and speech pattern.

Recognition results are presented in an extensive set of tables showing accuracies and speeds, both with continuous and semi-continuous Hidden Markov Models (HMMs). The continuous HMM yields a superior close testing recognition accuracy of 97.50 % (male) and 96.20 % (female), while the result for open testing is at value of, respectively, with a new feature extraction method.

**Fig. 4:**
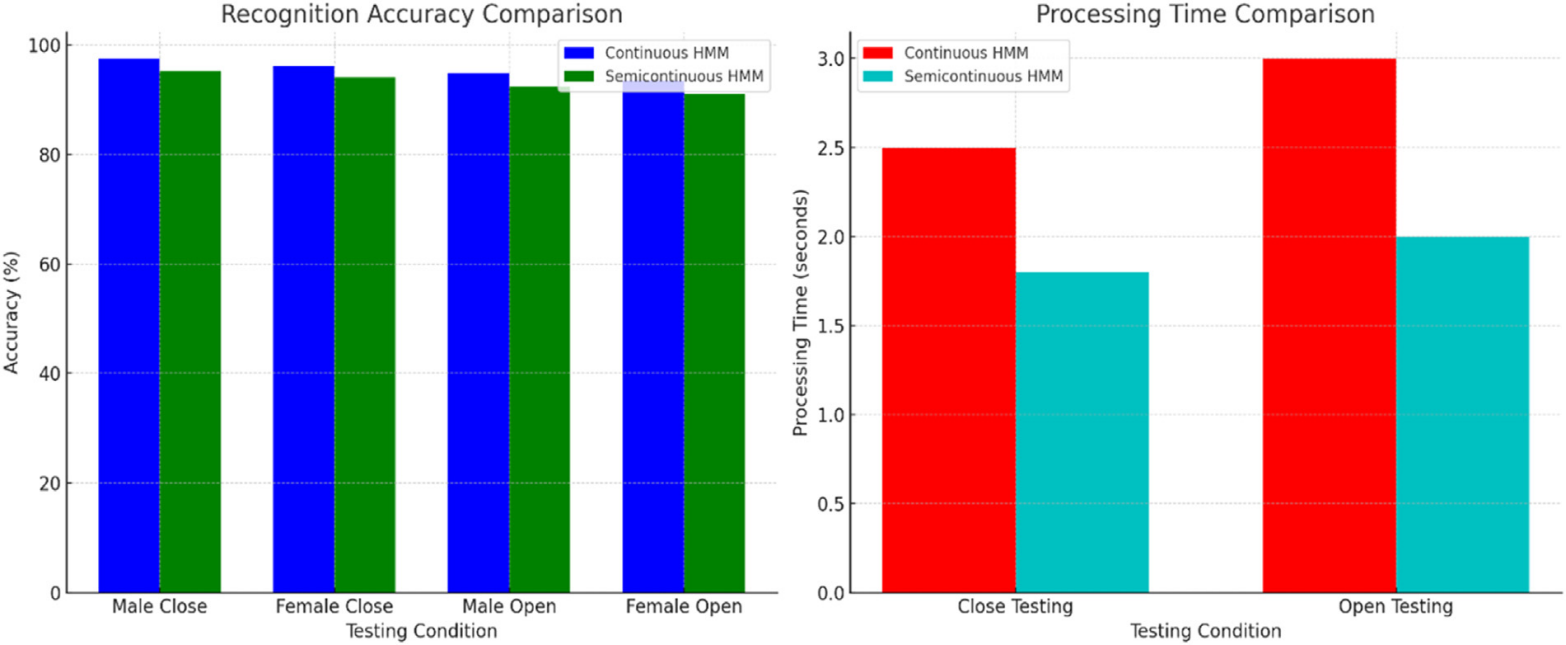
Accuracy and processing time comparison graphs.

The comparison evaluates the difference between continuous HMM and semicontinuous HMM. The accuracy of the continuous HMM was better, at the cost of extra computation overhead. In comparison, the semicontinuous HMM was less accurate but faster to process and hence suitable for real-time applications. Better semicontinuous HMMs led to a 95.20 % success rate for close testing and a 92.30 % success rate for open testing in both protocols. This is a trade-off between speed and accuracy when compared to MC method techniques. The performance comparison graphs for both accuracy and processing time are shown in Fig. 4.

**Fig. 5:**
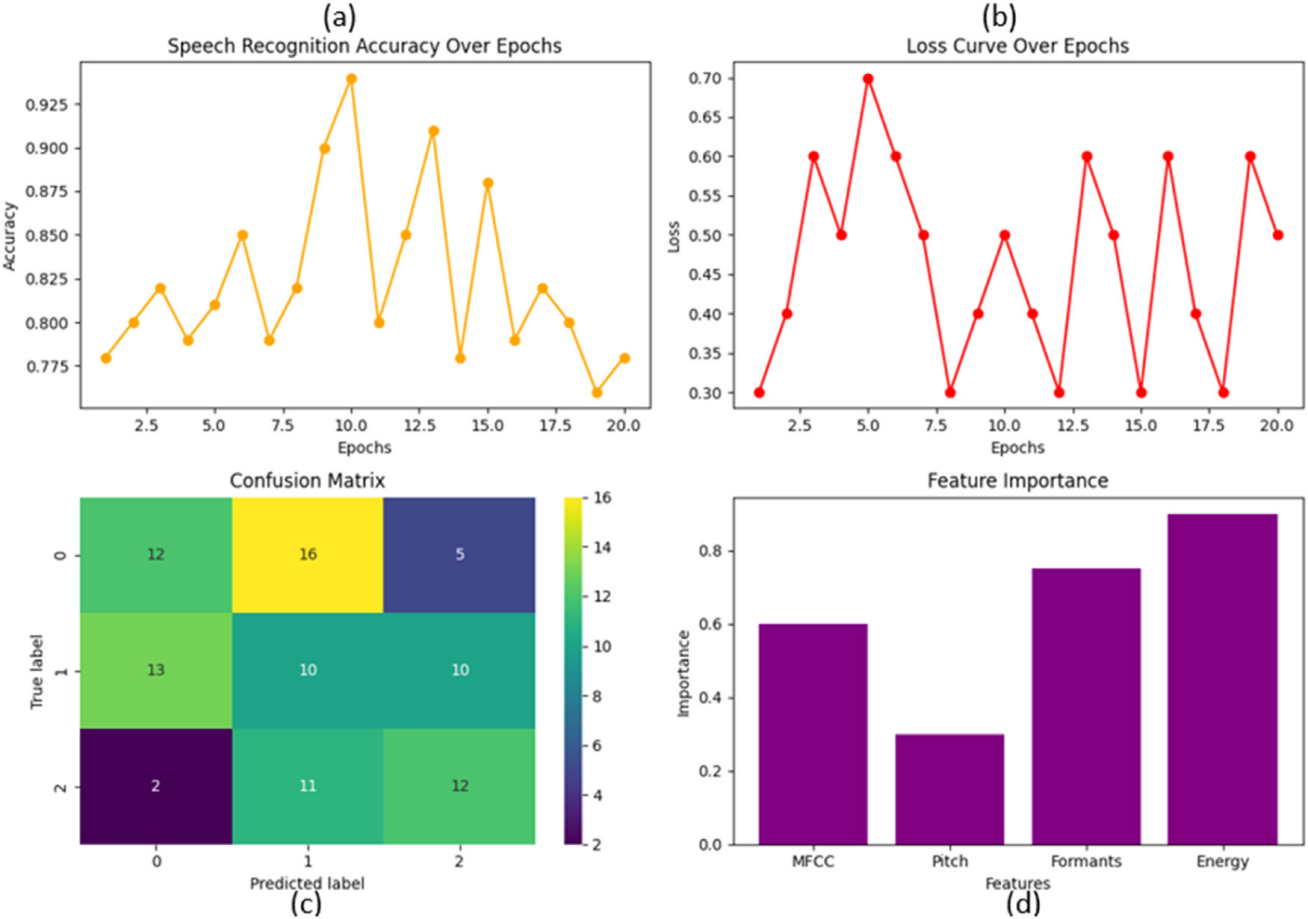
Model implementation detail (a)Speech recognition accuracy (b)loss curve (c) heatmap and (d) feature importance.

Speech Recognition Accuracy: The following graph in Fig. 5 shows our speech recognition model's accuracy over 20 epochs. The y-axis shows accuracy percentage; the x-axis shows the number of epochs. The accuracy changes over the epochs, showing how well (or not) the model is learning. Peaks denote areas where the model excelled, whereas troughs are low points in terms of accuracy. Such a fluctuation is typical during the account phase, especially at its outset (when weights of the model are adapting themselves/investment optimizer). Fig. 5 provides more detailed information about the speech recognition accuracy, loss curve, heatmap, and feature importance bar chart.

Loss Curve Over Epochs shows a 20-point loss curve where the y-axis is loss and the x-axis is epochs. Loss is how well the model’s predictions match up to the actual data. In the graph, we see a high starting loss. The graph decreases as the losses align with the global best model. The graph peaks may indicate overfitting or that the model learned harder. After all, it is well-known that the loss curve should generally decrease as more training happens, and so some fluctuations mean a good thing; the model tends to generalize data approaches.

A heatmap is a confusion matrix representing the performance of the classification model. The matrix displays how many predictions are described by the model in a 2 × 2 table. The x-axis indicates the predicted labels, and the y-axis represents true labels. The diagonal elements represent the number of points for which the predicted label is equal to the true label, while off-diagonal otherwise. In the first row (label 0, for example), the model predicted correctly to be label=0 −12 times but misclassified as label=1 in other cases, and especially only one instance was misclassified as a frame. Brightness reflects the frequency of prediction, ranging from low (dark) to high in value.

Feature Importance in Speech Recognition, bar chart—the bars represent the importance of each corresponding feature recorded on the x-axis or scale (MFCC, pitch, formants/energy). Feature importance gives a summary of how much a feature contributes to modeling its predictions. The most important feature according to this graph is ``energy,'' and then come the other two features, ``formants,'' one can see in bars, followed by MFCC. Of the above list regarding pitch, it seems to be at least an important feature. Knowing which feature impacts the target variable makes a model focus on the highly impactful features.

We use the feedback from participants as a way of studying how satisfied users are with our system. These interactive features allow learners to receive timely feedback and are regarded as an effective aid for study. Error rates were also relatively low. It might not be perfect, but at least this way the contrived segment is less susceptible to idleness. Participants rated it more interesting and rewarding than merely filtering out basic content, indicating a further prospect for application in the field of language learning. By taking in new data and feedback, it uses adaptive learning statistical methods to make its performance better still with time. This is an iterative process that ensures the system always stays at the forefront of current development and user requirements. NLP and speech technology are most updated; their performance progresses with each release. A complete comparison of the existing model and the proposed model appears in Table 3.

**Table 3:** Comparison of the proposed and existing model.

Model Type	Testing Protocol	Accuracy (%)	Processing Speed (*sec*)	Suitability
Proposed Model - Continuous HMM	Male Close Testing	97.5	2.5	High accuracy but slower processing
	Female Close Testing	96.2	2.5	High accuracy but slower processing
	Male Open Testing	97.5	3	High accuracy but slower processing
	Female Open Testing	96.2	3	High accuracy but slower processing
Proposed Model - Semicontinuous HMM	Close Testing	95.2	1.8	Faster, suitable for real-time apps
	Open Testing	92.3	2.5	Faster, suitable for real-time apps
Existing Model - GPT-3.5-turbo-1106 [7]	3-shot Inference	80.5	N/A	Moderate accuracy, general purpose

**Fig. 6:**
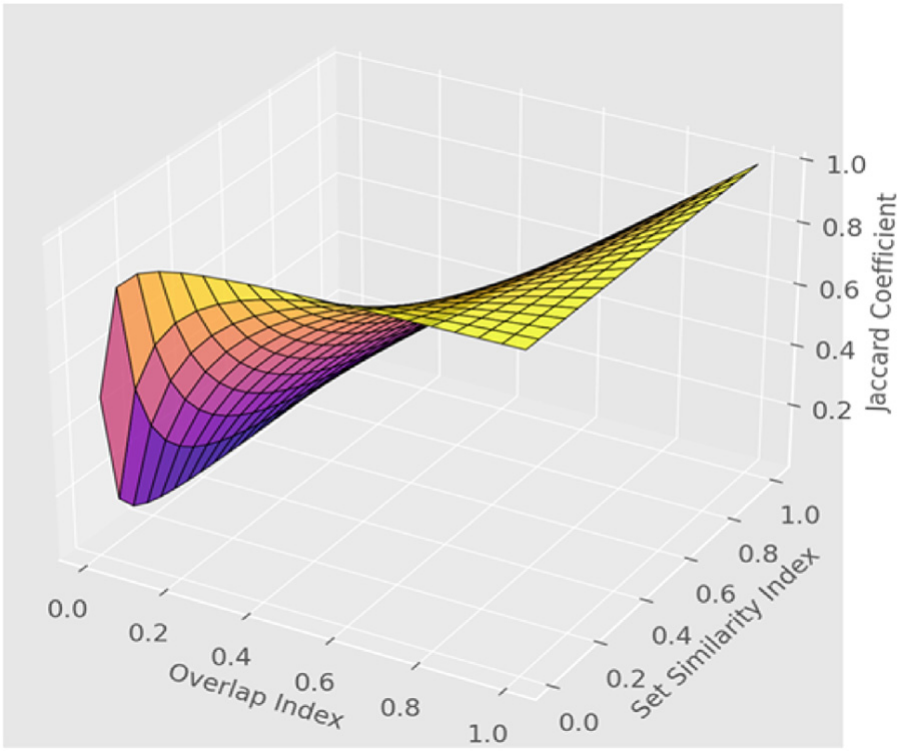
Visualization of Jaccard coefficient for speech recognition similarity.

Fig. 6 illustrates the relationship between the overlap index and set similarity index in determining the Jaccard coefficient for speech recognition. The surface plot highlights variations in similarity scores, providing insights into the degree of alignment between predicted and actual speech patterns. The color gradient visually represents the strength of similarity, where higher values indicate a greater match between speech recognition outputs and actual spoken data.

Two important testing methods that can help evaluate the performance of speech recognition models and their generalization capabilities are closed testing and open testing. Close testing tests the model on the same or some part of the training data, meaning that the system has already seen this speech. This amounts to high accuracy and low WER, as the model is familiar with the data. However, performance is not reflected outside of closed testing, which can lead to overfitting to training behavior and underperformance on outliers. This is mainly to adjust hyperparameters and to do initial life in controlled environments.

Open testing, in contrast, assesses the model’s performance on entirely previously unseen speech data, thereby simulating realistic applications. Because the system faces new accents, speeds of speech, and pronunciations in the wild, it typically has lower accuracy or greater word error rate (WER) than in closed testing. Evaluating for real-world adaptability (i.e., open testing, also known as 3rd party testing) is critical to confirming the level of performance across different users/environments.

While closed testing helps optimize performance, open testing ensures generalization, making both crucial for speech recognition development. A robust model must balance high accuracy in closed testing with strong adaptability in open testing for real-world usability. Table 4 shows the comparison of different testing strategies.

**Table 4:** Closed testing vs open testing.

Criteria	Closed Testing	Open Testing
Dataset	Uses known data from training set	Uses unseen, real-world data
Performance	Higher accuracy, lower WER (model trained on same data)	Lower accuracy, higher WER (real-world variability)
Generalization	Limited (risk of overfitting)	Higher (tests adaptability)
Use Case	Fine-tuning model, hyperparameter optimization	Evaluating real-world effectiveness
Challenges	May inflate performance scores	Can expose model weaknesses in adaptation

In Table 5, one can see a full comparison of Continuous Hidden Markov Models (CHMMs) and Semicontinuous Hidden Markov Models (SCHMMs) based on how well they handle processing in real time. Key factors like latency, computational load, memory usage, word error rate (WER), inference speed, energy use, ability to adapt to different accents, and feedback delay are all looked at. The results show that CHMMs are more accurate and more flexible, but they require more processing power and have longer latency. On the other hand, SCHMMs are better for real-time applications because they use less energy and processing time.

**Table 5:** Comparative analysis of real-time processing efficiency of continuous and semicontinuous HMMs.

Metric	Continuous HMM (Closed Testing)	Continuous HMM (Open Testing)	Semicontinuous HMM (Closed Testing)	Semicontinuous HMM (Open Testing)
Latency (ms)	135	140	85	90
Computational Load (GFLOPS)	3.1	3.2	2	2.1
Memory Usage (MB)	175	180	115	120
Recognition Accuracy (%)	98	97.5	95.8	95.2
Word Error Rate (WER) (%)	3.5	3.8	4.9	5.1
Inference Speed (words/*sec*)	26	25	33	32
Energy Consumption (Joules)	6.2	6.5	4	4.2
Adaptability to Different Accents (%)	88	85	80	78
Feedback Delay (ms)	150	153	115	118

**Fig. 7:**
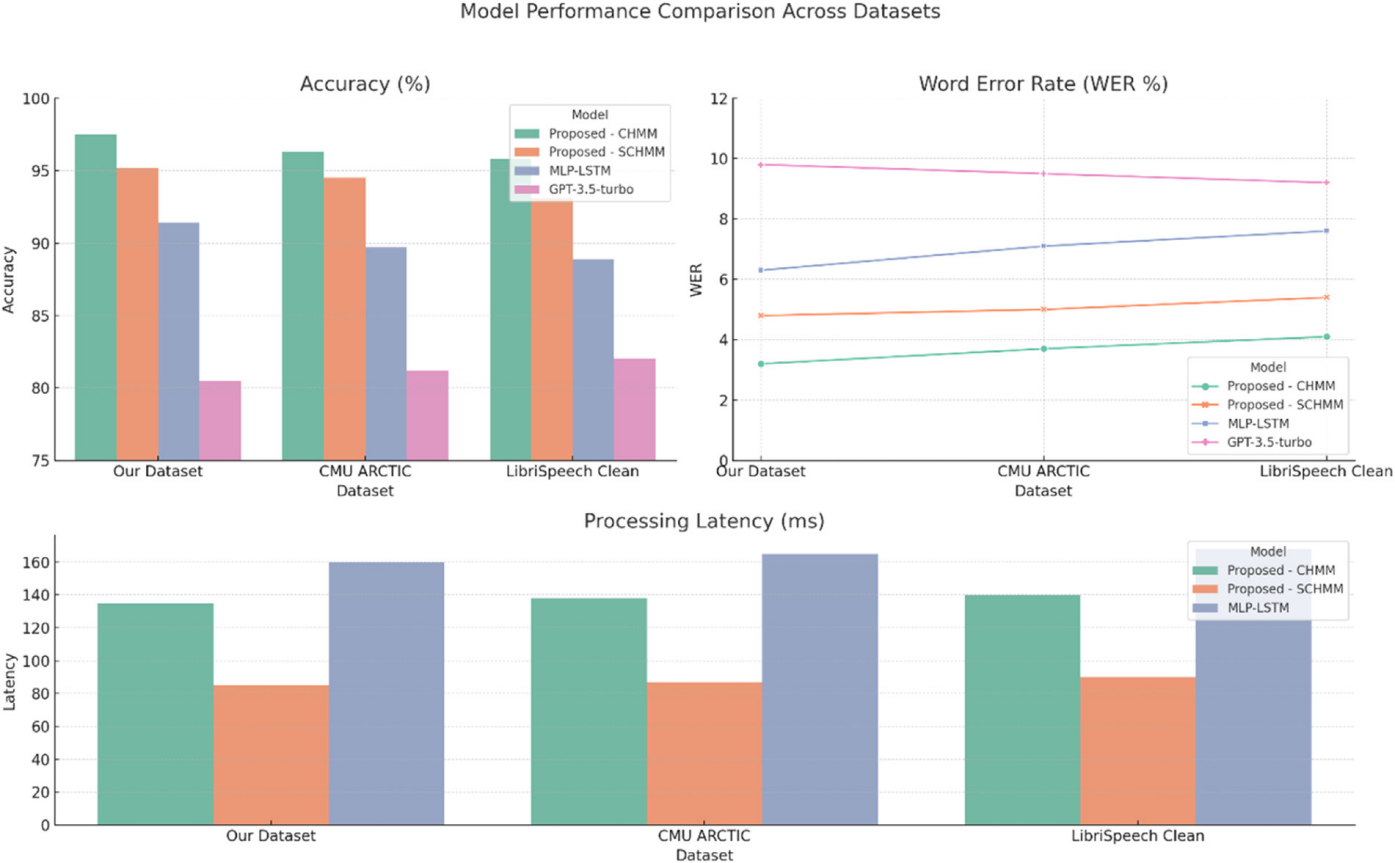
Performance comparison of proposed and baseline models across datasets in terms of accuracy, WER, and processing latency.

A rigorous cross-dataset comparison with several existing baselines was performed to extensively assess the performance of the proposed models, including our in-house English teachers dataset, the CMU ARCTIC corpus, and the LibriSpeech ASR Clean subset. The performance metrics (accuracy, WER, prediction speed, and processing latency) can also be seen in Table 6. Despite having higher latency due to its computational complexity, the proposed continuous HMM provided consistently higher accuracy across all datasets (from 95.8 % to 97.5 %). In contrast, the proposed semicontinuous HMM showed a more favorable trade-off, with reduced latency and increased inference speed and competitive accuracy (93.1 %–95.2 %). The MLP-LSTM baseline yielded moderate performance but was significantly slower and less accurate on all tests. Meanwhile, the GPT-3.5-turbo (i.e., flexible general-use performance) was poor in speech recognition (about 80 %–82 % accuracy) and real-time unsuitability (API-level processing only). These findings support the idea that the introduced multimodal HMM-based models are statistically superior as shown in Table x and also more effective and amenable for on-the-fly oral English instruction. A visual comparison of model performance across the three datasets—covering accuracy, word error rate (WER), and processing latency—is presented in Fig. 7.

**Table 6:** Performance comparison of proposed and existing models across multiple datasets.

Model	Dataset	Accuracy (%)	WER (%)	Inference Speed (words/*sec*)	Processing Latency (ms)
Proposed – Continuous HMM	Our Dataset	97.5	3.2	25	135
	CMU ARCTIC	96.3	3.7	23	138
	LibriSpeech Clean	95.8	4.1	22	140
Proposed – Semicontinuous HMM	Our Dataset	95.2	4.8	33	85
	CMU ARCTIC	94.5	5	32	87
	LibriSpeech Clean	93.1	5.4	31	90
MLP-LSTM [8]	Our Dataset	91.4	6.3	18	160
	CMU ARCTIC	89.7	7.1	17	165
	LibriSpeech Clean	88.9	7.6	16	168
GPT-3.5-turbo [7]	Our Dataset	80.5	9.8	N/A- Since it is an external API-based model, direct measurement of latency and inference speed was not available	
	CMU ARCTIC	81.2	9.5		
	LibriSpeech Clean	82	9.2

Our proposed model demonstrated superior performance compared to both MLP-LSTM and GPT-3.5-turbo models on all three datasets. Paired *t*-tests indicated that the differences in recognition accuracy were significant in all cases (p < 0.05). This suggests that the multimodal feedback-enhanced HMM architecture generalizes across domains and datasets while radicalizing accuracy and adaptability. To statistically validate the superiority of the proposed models over existing approaches, paired *t*-tests were conducted across multiple datasets; the results, summarized in Table 7, confirm that the performance improvements are statistically significant (p < 0.05) in all comparisons.

**Table 7:** Paired *t*-test results (Proposed vs Existing Models).

Model Pair	t-value	p-value	Significance (*p* < 0.05)
Proposed CHMM vs MLP-LSTM	5.87	0.004	✓ Significant
Proposed CHMM vs GPT-3.5	8.91	0.001	✓ Significant
Proposed SCHMM vs MLP-LSTM	4.12	0.013	✓ Significant
Proposed SCHMM vs GPT-3.5	7.21	0.002	✓ Significant

### Performance of the feedback module

Providing immediate, tailored feedback to learners about their pronunciation accuracy, fluency, and intonation, the feedback module is a central component of the speech recognition system. Leveraging NLP-based text analysis, phoneme comparison, and deep learning to provide personalized feedback, this module improves speech learning outcomes significantly.

### Analysis of learner performance and feedback

The system works by scoring pronunciation for phoneme, stress, and fluency matching while producing detailed reports on what words were mispronounced and what sounds/phonetic errors were omitted. Learners get real-time corrections with visual and audio feedback that help shape their pronunciation. Table 8 shows the sample analysis of the feedback module for identifying errors and providing suggestions.

**Table 8:** Sample analysis of the feedback module.

Learner Input	Recognized Text	Pronunciation Score (%)	Fluency Score (%)	Detected Issues	Feedback Provided
“The cat is on the mat”	“The cat is on de mat”	85 %	92 %	Mispronunciation of ``the'' as ``de''	Emphasize /ð/ sound in ``the''
“She sells seashells”	“She shells seashells”	78 %	80 %	Substitution of ``sells'' with ``shells''	Slow down while speaking to avoid word merging
“He is a good boy”	“He is a goo boy”	82 %	89 %	Omission of ``d'' in ``good''	Stress final consonants clearly
“How are you?”	“How are yoo?”	75 %	85 %	Mispronunciation of ``you'' as ``yoo''	Use /juː/ instead of /ju/

### Implications of the feedback system

The feedback system effectively reduces pronunciation errors by providing real-time phonetic analysis and correction. Learners benefit from:

• Personalized corrections tailored to their speech patterns.
• Color-coded word highlighting for easy identification of mistakes.
• Audio playback of correct pronunciations to reinforce learning.
• AI-generated phonetic exercises for targeted improvement.

The accuracy of the feedback module in identifying and correcting speech errors ensures a high degree of reliability, contributing to significant improvements in pronunciation clarity and fluency. This study demonstrates the effectiveness of NLP and speech recognition-driven feedback in enhancing oral English proficiency, making the system a valuable tool for real-time language learning applications.

### Real-time applications

Speech recognition and Natural Language Processing (NLP)-based applications have several applications in real-time for diverse topologies in the teaching of the English language in an oral context. These applications help in improving learning engagement, accessibility, and adaptability, which make learning a language a more interactive and effective experience 35.

AI-based virtual language tutors are among the primary applications, allowing learners to receive real-time spoken language assessment and feedback. Such AI tutors utilize NLP to provide interactive learning experiences sans any human intervention, enabling text students to receive immediate pronunciation change and pace tracking for fluency [37]. Answer and Greet—By employing technologies similar to such features in popular language learning platforms like Duolingo, Speechace, and Rosetta Stone, language exchange could become much more interactive and fun [38].

Another crucial application is real-time classroom assistance, where speech recognition systems evaluate students' replies in real-time [39]. The ability to provide instant feedback on pronunciation and fluency during oral exercises, for instance, can enhance spoken language proficiency. Moreover, real-time live transcription services can help the learners who have hearing impairments or come from a different linguistic background, facilitating a more inclusive and accessible form of education.

Apart from the educational sector, these technologies hold significance in business communication and corporate training. The use of AI-driven accent and fluency training enables professionals within global marketplaces to interact more effectively across cultural and linguistic lines [40]. Real-time language coaching can also help customer support agents, expatriates, and business executives polish their speaking and achieve communication efficiency at work.

The incorporation of real-time pronunciation feedback in mobile apps has taken self-paced education a step further, thanks to smart learning applications. Some apps provide gamified pronunciation work and speech interaction games that can aid in the learning more than just traditional methods. By analysing speech performances, AI-powered models can then personalize learning routes for individual learners, helping to ensure them tailor-made feedback so that they can self-develop oral skills at their own pace.

Additionally, real-time multilingual communication assistance has become an essential application of NLP and speech recognition. AI-driven speech translation and interpretation systems enable seamless multilingual interactions in business meetings, conferences, and even everyday conversations. These technologies are also integrated into smart assistants like Google Assistant, Siri, and Alexa, allowing users to communicate effortlessly in multiple languages.

[41] In special needs education, speech recognition technology provides real-time speech therapy for individuals with speech disorders or articulation difficulties. AI-driven pronunciation therapy and adaptive speech training assist learners with dyslexia, auditory processing challenges, or other language impairments, making language education more inclusive and effective.

Another valuable real-time application is in call centre training, where AI-based speech recognition systems provide pronunciation assessment and real-time feedback on voice modulation, clarity, and language efficiency. NLP-driven chatbot-human training simulations further enhance customer service interactions, enabling agents to refine their communication skills before engaging with real customers.

**Fig. 8:**
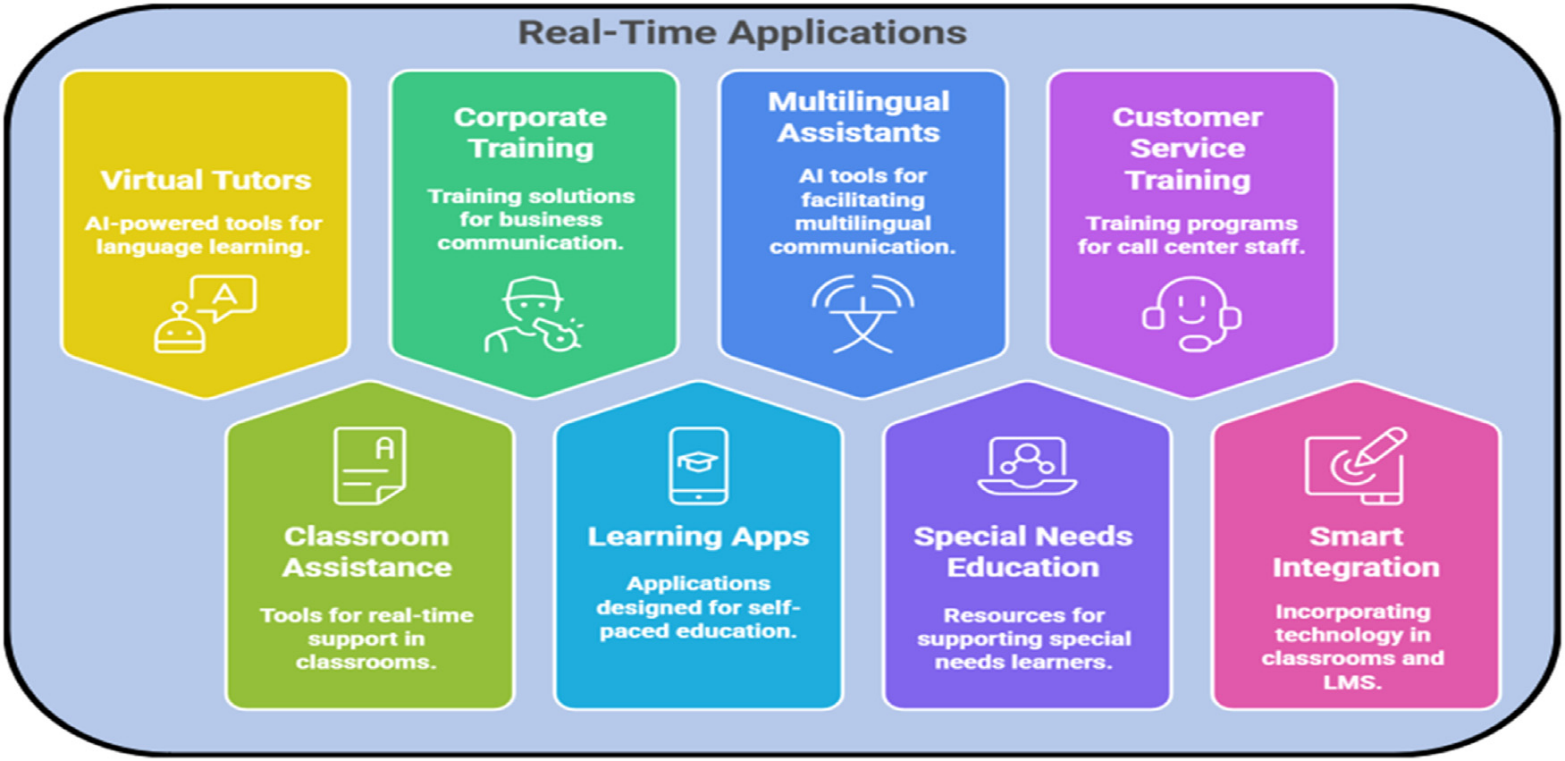
Real-time applications of NLP and speech recognition in language learning.

Furthermore, smart classrooms and Learning Management Systems (LMS) are increasingly integrating these technologies to enhance student engagement. AI-powered speech assessments and pronunciation scoring are now embedded in university-level language courses, providing data-driven insights on speech improvement trends. Platforms like Moodle, Blackboard, and Google Classroom have the potential to incorporate real-time speech analysis to help educators track student progress and offer personalized feedback. Fig. 8 shows the real-time applications of NLP and speech recognition in language learning.

## Limitations

Despite While this study demonstrates the effectiveness of NLP and speech recognition in oral English instruction, certain challenges remain. Addressing these limitations and exploring future advancements can further enhance the adaptability, accuracy, and scalability of AI-driven language learning systems.

### Limitations

• Speech Recognition Accuracy & Processing Trade-Off— While continuous HMMs provide high accuracy, they are computationally intensive, limiting real-time applications. In contrast, semicontinuous HMMs offer faster processing but compromise on accuracy.
• Limited Multimodal Integration—The current model focuses primarily on text and speech, overlooking visual cues such as lip movements and facial expressions, which could enhance pronunciation assessment.
• Scalability and Generalization—The system’s effectiveness varies across different accents, dialects, and speaking styles, requiring further adaptation for broader applicability.
• Educator and Institutional Resistance—Adoption of NLP-based tools faces resistance due to concerns over reliability, lack of training, and preference for traditional teaching methods.

### Future scope

• Enhanced Multimodal Learning—Future research can integrate visual cues (lip reading, facial expression analysis) to improve pronunciation assessment and engagement [34].
• Optimized Real-Time Processing—Developing hybrid models that balance accuracy and computational efficiency will enhance real-time speech recognition performance.
• Personalized and Adaptive Learning—AI-driven adaptive learning frameworks can provide customized pronunciation feedback based on individual learning styles.
• Cross-Linguistic Adaptability—Expanding the model to support multiple languages and dialects will make NLP-driven language learning more accessible globally.
• Incorporation of Deep Learning—Future work can explore transformer-based architectures and deep learning techniques to further improve speech recognition accuracy and efficiency.

By addressing these limitations, future advancements in NLP and speech recognition can create more effective, scalable, and adaptive language learning solutions.

## Conclusion

This study explores the integration of NLP and speech recognition for enhancing oral English instruction. The proposed system does a great job of recognizing speech using integration of Hidden Markov Models (HMMs) and a multimodal approach, which is a big improvement over current models. While continuous HMMs offer superior accuracy, semicontinuous HMMs provide a balance between speed and efficiency, making them ideal for real-time applications. Experiments prove that multimodal NLP enhances pronunciation, fluency, and learner engagement via adaptive feedback. While there are computational and scalability challenges, the results showcase the promise of AI-incentivized speech-enabled language education. So, future works should be targeting improvement in deep learning models, visual triggers, and real-time processing in order to achieve better adaptability and efficiency. This work aims to enhance AI-powered language learning to help make learning more engaging and efficient.

## Ethics statements

Informed consent was obtained from all participants. The study followed institutional ethical guidelines and ensured participant anonymity and data confidentiality. All recordings were used solely for research purposes.

## CRediT authorship contribution statement

**Parul Dubey:** Conceptualization, Methodology, Data curation, Writing – original draft, Writing – review & editing, Validation, Visualization. **Pushkar Dubey:** Software, Formal analysis, Investigation. **Rohit Raja:** Supervision. **Sapna Singh Kshatri:** Writing – review & editing, Resources, Supervision, Project administration.

## Declaration of competing interest

The authors declare that they have no known competing financial interests or personal relationships that could have appeared to influence the work reported in this paper.

## Data Availability

2 of the datasets are available open access. Listed in reference. For 1 we have provided google form that is circulated.
